# Tofacitinib is an effective treatment for moderate to severe ulcerative colitis, and intestinal ultrasound can discriminate response from non-response: a pragmatic prospective real-world study

**DOI:** 10.1080/07853890.2024.2358183

**Published:** 2024-05-30

**Authors:** Jacob E. Ollech, Hagar Eran-Banai, Idan Goren, Tali Sharar Fischler, Irit Avni-Biron, Yifat Snir, Yelena Broitman, Shaked Cohen, Adi Friedenberg, Maor H. Pauker, Iris Dotan, Henit Yanai

**Affiliations:** a Division of Gastroenterology, Rabin Medical Center, Petah-Tikva, Israel, Faculty of Medicine, Tel Aviv University, Tel Aviv, Israel; b Department of Inflammation and Immunity, Lerner Research Institute, Cleveland Clinic, OH, U.S.A

**Keywords:** Intestinal ultrasound, ulcerative colitis, tofacitinib

## Abstract

**Introduction:**

Real-world data on tofacitinib’s effectiveness is limited and mainly retrospective or registry-based. We elected to conduct a pragmatic prospective study to assess the efficacy of tofacitinib for moderate to severe ulcerative colitis (UC), aiming to evaluate the ability of intestinal ultrasound (IUS) to discriminate responders vs. non-responders in real-time.

**Methods:**

This pragmatic prospective clinical study included consecutive adult patients starting tofacitinib treatment for active moderate to severe UC. Patients were evaluated at baseline and after 8 weeks of tofacitinib (clinical, biomarker, endoscopy, and IUS). The primary outcome was clinical response defined by a decrease in the full Mayo score (fMS) of ≥3 at week 8. Next, we explored ultrasonographic parameters in the sigmoid colon as potential real-time classifiers to differentiate between responders and non-responders at week 8.

**Results:**

Overall, 30 adult patients started tofacitinib; the median age was 26.3 years (IQR 22.5–39.8), and 50% were female. Most patients (86.6%) had left-sided or extensive colitis, 96.7% had previously failed biologic therapy, and 60% (18/30) were on oral corticosteroids at the start of tofacitinib. At week 8, clinical response (a decrease in the fMS ≥ 3) and remission (fMS ≤ 2) rates were 40% (12/30) and 20% (6/30), respectively. Biomarker response (FC < 250µg/g) and biomarker normalization (FC ≤ 100µg/g) were achieved in 47.6% (10/21) and 38.1% (8/21) of patients, respectively. Endoscopic healing (endoscopic Mayo sub-score [EMS] ≤ 1) was achieved in 33.3% (10/30) of patients. Sigmoid bowel wall normalization as assessed by IUS (sBWT ≤ 3) was achieved in 18.2% (4/22). The best sBWT cut-off at week 8 to accurately classify endoscopic healing vs. no healing was a sBWT of 3.6 mm (AUC of 0.952 [95% CI: 0.868–1.036], *p* < 0.001).

**Conclusion:**

In this real-world pragmatic prospective study, tofacitinib was an effective treatment for moderate to severe UC, and IUS at week 8 accurately discriminated treatment response from non-response.

## Introduction

Ulcerative colitis (UC) often leads to disability, complications, and reduced quality of life. While biologic therapies have improved outcomes for some patients, many still experience treatment failures [1,2], highlighting the need for effective and safe medications with alternative mechanisms of action [3].

Tofacitinib, a Janus kinase (JAK) inhibitor, has shown promise in randomized controlled trials (RCTs) for inducing and maintaining remission in moderate to severe UC. [4]. However, RCTs’ strict inclusion and exclusion criteria may not reflect real-world patient populations, necessitating real-world effectiveness studies. Current real-world data on tofacitinib’s effectiveness in UC are limited and mainly retrospective or registry-based [5–13]. Although guidelines recommend assessment of response to therapy with endoscopy [3], in actual practice, timed early endoscopy may be less feasible, as it is invasive, labour-intensive, costly, and poorly tolerated by patients [14].

We conducted a real-world pragmatic study to assess tofacitinib’s effectiveness for moderate to severe UC. Intestinal ultrasound (IUS) is an easy-to-use tool to evaluate activity in UC and response to therapy [15,16]. This study aimed to prospectively evaluate the ability of IUS to discriminate clinical and endoscopic improvement from non-improvement after 8 weeks of tofacitinib therapy (induction period).

## Methods

### Study design and patients

This was a prospective pragmatic longitudinal single-center study among patients receiving tofacitinib in treating moderate to severe UC in adult patients (≥18 years of age). This study was conducted at a tertiary referral center (Rabin Medical Center, Israel) between September 2020 and April 2023. The decision to use tofacitinib was based on the physician’s discretion. All patients prescribed tofacitinib for active UC who agreed to participate in the study and signed an informed consent form were included. Patients who were prescribed tofacitinib for any other reason were excluded. The patients were given tofacitinib 10 mg twice daily for induction. Concomitant corticosteroids and mesalamine (5-aminosalicylic acid [5-ASA] regiments) (oral and topical) were allowed. At the start of the study (week 0) and at the end of the induction period (week 8), patients underwent a comprehensive assessment, which included clinical, biomarker, endoscopy, and IUS assessments. There was no forced corticosteroid tapering strategy. Adverse events were recorded throughout the study.

### Procedures

#### Clinical and endoscopic assessment

A clinical visit with full laboratory evaluation, including fecal calprotectin (FC), was conducted at baseline and the end of the induction. Enrollees were referred for endoscopy by either a complete colonoscopy or a flexible sigmoidoscopy at both time points, and biopsies were taken from the sigmoid and rectum. Clinical status was evaluated by the full Mayo score (fMS [stool frequency, rectal bleeding, endoscopic activity, and physician assessment]) [17]. The endoscopic activity was assessed by the endoscopic Mayo sub-score (EMS): 0 = normal or inactive disease; 1 = erythema, decreased vascular pattern, mild friability; 2 = absent vascular pattern, erosions; and 3 = spontaneous bleeding, ulcerations. Biopsy slides were read by gastrointestinal pathologists and scored with the Nancy index[18].

#### Intestinal ultrasound assessment

IUS was performed by a single gastroenterologist expert in IUS (H.B.E, > 5 years of experience), using a General Electric LOQIC E9 machine with convex (1-6MHz) and linear (3–8MHz) transducers. IUS was performed at both time points for patients with left-sided and pancolitis (patients with proctitis were excluded from the IUS analysis as the transabdominal IUS is limited in evaluating the rectum). Before the procedure, patients were not required to fast and did not receive any bowel preparation. Bowel wall thickness (BWT) was measured in the sigmoid colon (sBWT). Vascularization of the bowel wall was assessed with colour doppler, with the velocity scale set to 7 cm/s to maximize the detection of small vessels within the bowel wall. The mucosa was identified as the hypoechoic layer between the lumen and submucosa. Submucosa was identified as the hyperechoic layer between mucosa and muscularis propria. Muscularis propria was identified as the hypoechoic layer between the submucosa and the serosa. Hypervascularization (colonic wall flow) was graded using the modified Limberg score, including four categories: [0] absent, [1] small spots within the wall, [2] long stretches within the wall, and [3] long stretches within the wall extending into the mesentery [19]. Additionally, the Milan ultrasound criteria (MUC), which is a validated score developed to assess endoscopic activity in UC (MUC = 1.4 X bowel wall thickness in the sigmoid colon +2.0 X bowel wall flow [present = 1, absent = 0]) was ­calculated [20].

### Outcomes

The primary outcome was clinical response at week 8. Patients were also assessed for clinical remission, biomarker response and normalization, endoscopic healing, ultrasonographic bowel wall normalization, improvement in hypervascularization, and ultrasonographic remission by the MUC. Ultrasonographic parameters were assessed as potential classifiers for clinical response.

#### Definitions of clinical and endoscopic Endpoints

Clinical response was defined as a decrease in the fMS ≥ 3 and clinical remission as a fMS of ≤2 points. Biomarker response and normalization were defined by FC < 250 mg/kg and <100 mg/kg, respectively (this was assessed only for patients with FC > 250 mg/kg at baseline). Endoscopic healing was defined as an EMS ≤1.

#### Definitions of ultrasonographic Endpoints

Ultrasonographic bowel wall normalization was defined as BWT ≤ 3 mm in the sigmoid colon [^*15,21*^]. Improvement in hypervascularization was defined by a drop of 1 point in the modified Limberg score. Ultrasonographic remission by MUC was defined as a score ≤ 6.2[15]. These calculations were considered only in patients with abnormal baseline ultrasonographic measures.

### Statistical analysis

Descriptive statistics were presented as medians with an interquartile range (IQR) for continuous variables and percentages for categorical variables. Rates of the different efficacy endpoints were calculated for both the intention-to-treat (ITT) group (all patients who underwent baseline evaluation and started tofacitinib) and per-protocol (PP). Continuous variables were analysed using non-parametric testing with the Wilcoxon matched-pair signed-rank test or the Mann-Whitney test, as appropriate. Categorical variables were compared using the χ ^2^ test or Fisher exact test, as appropriate.

The Correlation between the exploratory IUS parameters (sBWT at 8 weeks, the change in sBWT within 8 weeks, and the MUC score at week 8) vs. the EMS and the fMS were evaluated with the Spearman’s correlation coefficient (rho). The ability of IUS parameters to classify clinical response vs. non-response at week 8 (response based on the fMS [binomial variable]) was analyzed with the receiver operating characteristic (ROC) plot. This was followed by the identification of the best cut-off values and calculation of the respective sensitivity, specificity, diagnostic accuracy, positive predictive value (PPV), negative predictive value (NPV), and likelihood ratios with a 95% confidence interval (CI).

A two-tailed *p* < 0.05 was considered statistically significant. The data were analyzed using IBM SPSS Statistics (Version 29) and GraphPad Prism version 10.0.

### Ethics, funding, and data availability

The institutional review board at Rabin Medical Centre approved the study protocol – reference number 0508-19. This study was funded by Pfizer and reports on an interim analysis of the patients who finished their evaluation during the induction period. The data underlying this article cannot be shared publicly due to the privacy of individuals who participated in the study. The data will be shared with the corresponding author upon reasonable request.

## Results

### Patients

We report on 30 patients who started tofacitinib to control active UC (included in the ITT analyses) of whom 27 patients finished the 8-week study. One patient discontinued tofacitinib after 4 weeks of therapy due to severe exacerbation, and two patients were lost to follow-up. Eighteen patients (60%) were on oral corticosteroids at the start of the tofacitinib, and 17 (56.7%) received 5-ASA formulations. The median patient age was 26.3 years (IQR 22.5-39.8); female (50%). Most patients (86.6%) had left-sided or extensive colitis, 29/30 patients (96.7%) had previously failed biologic therapy, and over a quarter failed two previous biologic therapies. At baseline, the median fMS was 8 (IQR: 7-10), and the median FC (*n* = 27) was 869 μg/g (IQR: 263-2100). See Table 1 for patient characteristics.

**Table 1.** Patient characteristics at baseline (*N* = 30, ITT).

**Variable at Baseline (week 0)*N* = 30** *intention to treat*	Count (%) or Median (IQR)
**Gender Female**	15 (50)
**Age (years)**	26.3 (22.5–39.8)
**Disease duration (years)**	3.43 (1.17–9.19)
**Smoking Status**	
*never*	26 (86.7)
*current*	3 (10)
*past*	1 (3.3)
**Disease Extent**	
*E1- Proctitis*	4 (13.3)
*E2- Left sided colitis*	16 (53.3)
*E3- Extensive colitis*	10 (33.3)
**Past Medications**	
5-ASA therapies	26 (86.7)
Corticosteroids	25 (83.3)
*Budesonide MMX*	12 (40.0)
*Prednisone*	23 (76.7)
IMM	11 (36.7)
Biologic therapy	29 (96.7)
*Adalimumab*	3 (10.0)
*Infliximab*	12 (40.0)
*Vedolizumab*	23 (76.7)
*Past > 1 biologic therapy*	8 (26.7)
**Concurrent Medications**	
5-ASA therapies	17 (56.6)
Corticosteroids	18 (60)
**Disease Activity**	
Full Mayo score (fMS)	8 (7-10)
Partial Mayo score	6 (4.7-8.0)
Endoscopic Mayo subscore (EMS)	2 (2-3)
Haemoglobin at baseline, g/dL	12.7 (11.4–14.7)
C-reactive protein, mg/L	0.48 (0.14–1.87)
Faecal calprotectin (FC), μg/g	869 (263–2100), *n* = 27
FC > 250 μg/g	21 (70)
Sonographic sigmoid colon bowel wall thickness (sBWT) (millimetre)	4 (3.4–4.5), *n* = 25
Modified Limberg score	2 (1–2), *n* = 25
The Milan ultrasound criteria (MUC)	7.46 (6.53–8.16), *n* = 25

IQR: interquartile range.; 5-ASA: 5 aminosalicylic acid; IMM: immunomodulator.

For the sonographic analysis we included 25patients (16 -left sided, 9 -extensive colitis), patients with proctitis were excluded.

### Efficacy outcomes

#### Clinical and endoscopic endpoints

Twenty-five patients had fMS at baseline (week 0) and the end of the induction (week 8) and were eligible for the PP analysis. At the end of the induction clinical response was achieved by 12/30 patients (40%) and 12/25 patients (48%) in the ITT and PP analyses, respectively. In the ITT and PP analyses, 6/30 patients (20%) and 6/25 patients (24%) achieved clinical remission. The fMS decreased from a median of 8 (IQR: 7–10) at baseline to 5 (IQR: 2.5–9.5) at the end of induction, *p* = 0.003. There were no differences in baseline characteristics between responders vs. non-responders at week 8; see Table 2.

**Table 2.** Comparing responders’ vs non-responders’ characteristics at baseline (*n* = 25, PPT).

Variable at Baseline (week 0) *n* = 25 *per-protocol*	Responders (week 8) *n* = 12 Count (%), or Median (IQR)	Non-responders (week 8) *n* = 13 Count (%), or Median (IQR)	p-value
**Gender Female**	6 (50)	6 (42.2)	0.848
**Age (years)**	24.9 (22.2–40.4)	27.1 (23.2–40.3)	0.689
**Disease duration (years)**	4.9 (1.11–10.6)	5.5 (1.5–13.4)	0.786
**Smoking Status**			
*never*	10	13	0.220
*current*	1	0	
*past*	1	0	
**Disease Extent**			
*E1- Proctitis*	0	3 (23.1)	0.324
*E2- Left sided colitis*	7 (58.3)	6 (46.2)	
*E3- Extensive colitis*	5 (41.7)	4 (30.8)	
**Past Medications**			
5-ASA	10 (83.3)	11 (84.6)	0.999
Corticosteroids	9 (75)	11 (84.6)	0.645
*Budesonide MMX*	6 (50)	6 (46.2)	0.848
*Prednisone*	8 (66.7)	10 (76.9)	0.673
IMM	5 (41.7)	5 (38.5)	0.999
Biologic therapy	12 (100)	12 (92.3)	0.999
*Adalimumab*	2 (16.7)	1 (7.7)	0.593
*Infliximab*	4 (33.3)	7 (53.8)	0.302
*Vedolizumab*	9 (75)	10 (76.9)	0.999
*Past > 1 biologic therapy*	2 (16.6)	6 (46.2)	0.202
**Concurrent Medications**			
5-ASA therapies	6 (54.5), *n* = 11	8 (66.7)	0.680
Corticosteroids	6 (50)	8 (66.7), *n* = 12	0.680
**Disease Activity**			
Full Mayo score (fMS)	8.5 (7–10.75)	7 (6.5–10)	0.611
Partial Mayo score	6 (5–7.75)	5 (3.5–7.5)	0.574
Endoscopic Mayo subscore (EMS)	3 (2–3)	2 (2–3)	0.538
Haemoglobin at baseline, g/dL	12.4 (11.4–12.8)	13.6 (12.0–15.4)	0.110
C-reactive protein, mg/L	0.33 (0.07–1.97)	0.56 (0.19–2.15)	0.406
Faecal calprotectin (FC), μg/g	1040 (265–1842)	869 (227–2415)	0.810
FC > 250 μg/g	10 (83.3)	10 (76.9)	0.999
Ultrasonographic sigmoid colon bowel wall thickness (sBWT), mm	4 (3.3–4.3), *n* = 11	4.4 (3.85–4.9), *n* = 10	0.085
Ultrasonographic sBWT >3 mm	11 (100), *n* = 11	10 (100), *n* = 10	0.999
Modified Limberg score	2 (1–2), *n* = 11	1.5 (0.75–2.25), *n* = 10	0.756
Ultrasonographic hypervascularization (positive colonic wall flow)	10 (90.9), *n* = 11	7 (70), *n* = 10	0.311
The Milan ultrasound criteria (MUC)	7.6 (6.62–8.02), *n* = 11	7.74 (7.03–8.64), *n* = 10	0.557
MUC > 6.2	10 (90.9), *n* = 11	9 (90), *n* = 10	0.999

IQR: interquartile range; 5-ASA: 5 aminosalicylic acid; IMM: immunomodulator.

For the sonographic analysis we included only patients left sided and extensive colitis.

Overall, 21 patients (70%) had an increased FC level ≥250 μg/g at baseline; biomarker response (FC < 250 μg/g) was achieved by 10/21 patients (47.6%), and biomarker normalization (FC < 100 μg/g) by 8/21 patients (38.1%). In this subgroup, the median decrease in faecal calprotectin was 994 μg/g (IQR: 210–1995), corresponding to 86.5% (IQR: 27.1%–95.8%), (*n* = 19 with paired data), *p* = 0.014.

**Figure 1.**
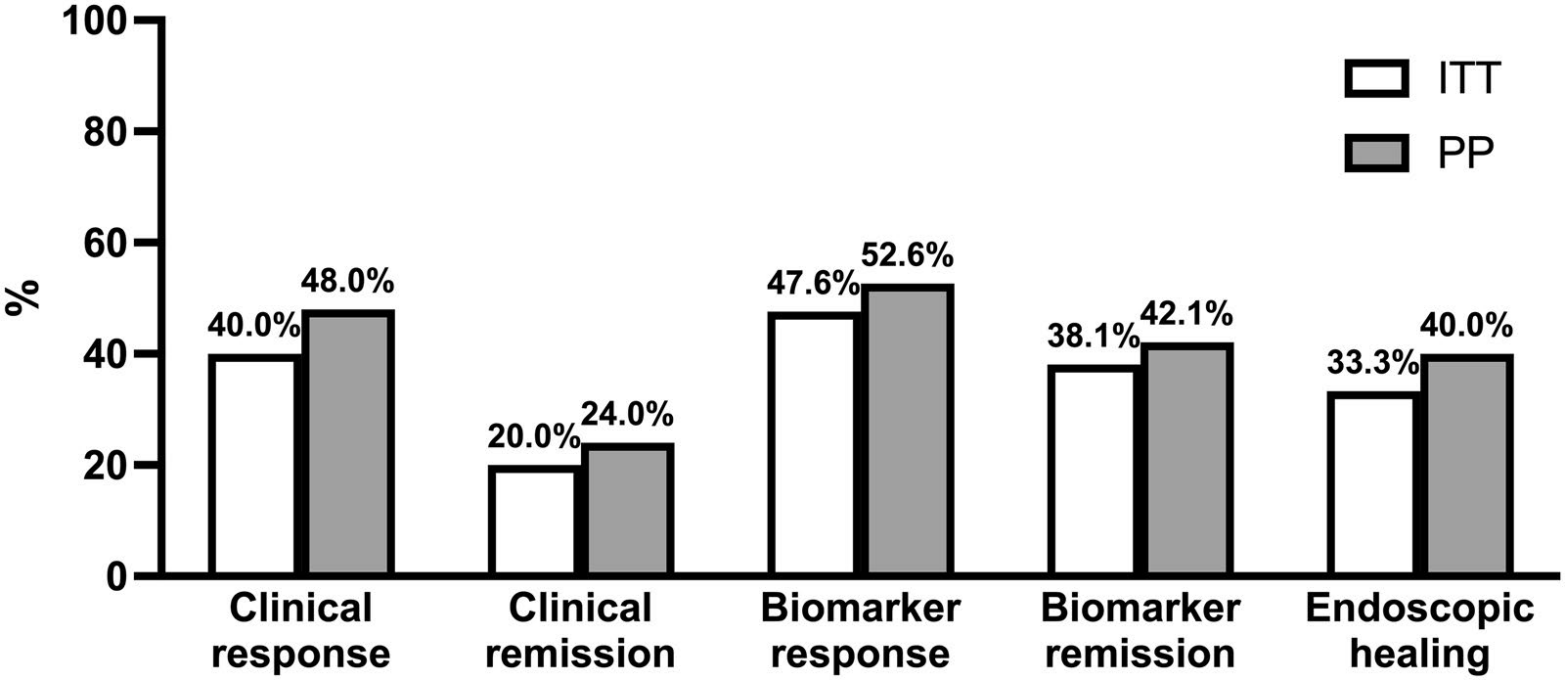
Clinical, endoscopic and biomarker response and remission rates at week 8.

**Figure 2.**
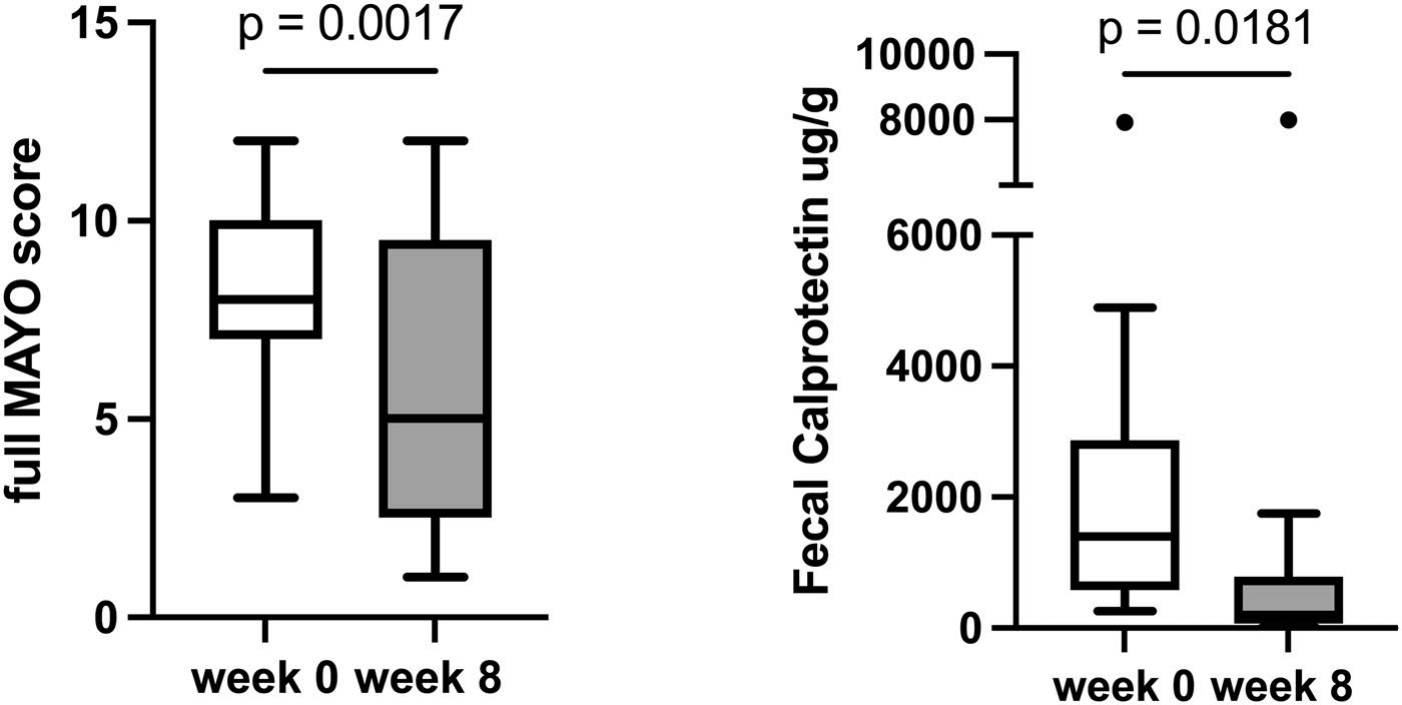
Changes in disease assessment parameters between weeks 0 and 8 among responders and non-responders.

Endoscopic healing was achieved by 10/30 patients (33.3%) and 10/25 patients (40%) in the ITT and PP cohorts, respectively. The median endoscopic score decreased from 3 (IQR: 2-3) to 2 (IQR: 1–2), *p* < 0.001. Twelve patients had paired histological samples; in this subgroup, the median Nancy score decreased from 3.5 (IQR: 3–4) to 1.5 (IQR: 1–3.75) over the 8-week induction period, *p* = 0.012. See Figures 1 and 2 for efficacy outcomes.

Of the 18 patients on corticosteroids at tofacitinib treatment start, 11 (63.1%) completely withdrew corticosteroids by the end of the induction, while 4 patients started corticosteroids within the induction period. Overall, at the end of the induction, 11 patients were still on corticosteroids (6/11 patients > 20 milligrams of prednisone), the vast majority had active disease (only one patient was in clinical remission while on 10 milligrams of prednisone). Eight patients were maintained on a combination of tofacitinib with 5-ASA formulations.

#### Intestinal ultrasound endpoints

Twenty-five patients underwent an IUS assessment at baseline (16 [64%] left-sided colitis, 9 [36%) extensive colitis). At baseline 22/25 patients (88%) had a sBWT > 3mm, 7/25 (28%) a sBWT> 4.43 mm, 21/25 (84%) a Limberg score ≥1 (positive for assessment of bowel wall hypervascularization), 18/25 (72%) had both sigmoid BWT> 3 mm and hypervascularization, and 20/25 (80%) patients a MUC >6.2.

The calculations of normalization/improvement were performed only for patients with abnormal values at baseline. Twenty-one patients had paired IUS conducted at both time points, enabling paired analysis of the dynamics in ultrasonographic parameters. Sigmoid bowel wall normalization (sBWT ≤ 3) was achieved in 4/22 patients (18.2%) and 4/20 patients (20%) in the ITT and PP analyses, respectively. Improvement in hypervascularization (a drop of 1 point in the modified Limberg score) in 12/21 patients (57.1%) and 12/17 patients (70.6%) in the ITT and PP analyses, respectively. Ultrasonographic remission by the MUC (a score ≤ 6.2) was evident in 8/20 patients (40%) and 8/18 patients (44.4%) in the ITT and PP analyses, respectively.

Within 8 weeks of tofacitinib therapy, the median SBWT decreased from 4.30 mm (IQR 3.65 − 4.60) to 3.50 mm (IQR 3.05 − 4.10), *p* = 0.030. The median drop in sBWT was 0.3 mm (IQR: 0.7 – [-0.05]), corresponding to a median reduction of 8.3% (IQR: 17.6% – [–1.2%]). This change in sBWT was more prominent among responders (*n* = 10) vs. non-responders (*n* = 10), revealing a median drop in sBWT of 0.4 mm (IQR: 2.3–0.2) vs. 0.05 mm (IQR: 0.45 – [–0.57]), corresponding to a median drop of 10.9% (IQR: 51.5–5.8) vs. 0.72% (IQR: 11.6% – [–11.1%]), respectively, *p* = 0.026. This overall decrease in the sBWT was associated with a reduction in the modified Limberg score from a median of 2 to 0 (*p* = 0.003) and a reduction in the median MUC score from 7.6 (IQR: 6.6–8.3) to 6.2 (IQR: 4.48–7.25), *p* = 0.011.

The correlations between the sBWT, the modified Limberg score, and the MUC with the EMS at week 8 were: rho 0.697, (95% CI: 0.368–0.871), *p* < 0.001, rho 0.488 (95% CI 0.058–0.766), *p* = 0.025, and rho 0.738 (95% CI: 0.438–0.890), *p* < 0.001, respectively. The correlations between the sBWT, the modified Limberg score, and the MUC with the fMS at week 8 were: rho 0.570, (95% CI: 0.170–0.808), *p* = 0.007, rho 0.435 (95% CI: [–0.01]–0.736), *p* = 0.049, and rho 0.620 (95% CI: 0.245–0.834), *p* = 0.003, respectively. There were no significant correlations between these parameters at week 0 and the EMS at week 0.

**Figure 3.**
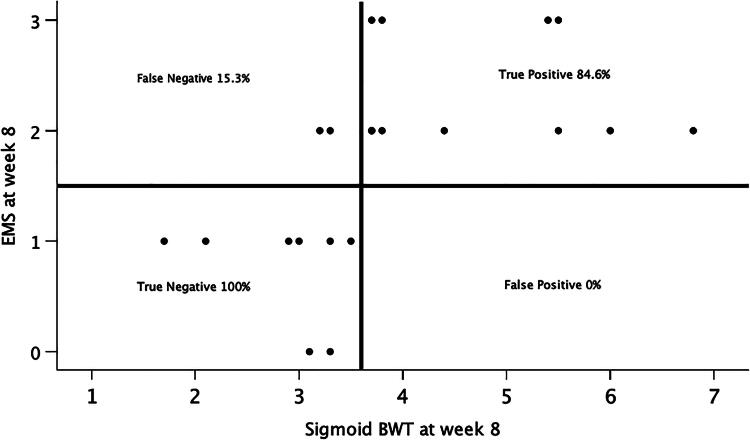
a sigmoid BWT cut-off of ≥3.6 mm at week 8 discriminates response from non-response at week 8. 3A – scattered plot to classify endoscopic healing vs. no healing (based on EMS ≤1); 3B – box plot to classify clinical response vs. no response (based on decreases in the fMS ≥ 3).

The best sBWT cut-off at week 8 to accurately classify endoscopic healing (EMS ≤ 1) vs. no healing and also clinical response (a decrease in the fMS ≥ 3) vs. no response at week 8 was a sBWT of 3.6 mm, yielding a sensitivity of 84.6% with a specificity of 100% for discriminating endoscopic healing at week 8 (AUC of 0.952 [95% CI: 0.868–1.036], *p* < 0.001), and a sensitivity of 90% with a specificity of 81.8% for discriminating clinical response at week 8 (AUC of 0.873 [95% CI: 0.714–1.031], *p* < 0.001). See Table 3 for the statistic metrics of sBWT cut-off ≥3.6 mm at week 8, and Figure 3 to demonstrate its ability as a discriminator.

**Table 3.** Statistic metrics of a sigmoid BWT cut-off of ≥3.6 mm at week 8 as a classifier.

3A. Statistic metrics of a sigmoid BWT cut-off of ≥3.6mm at week 8 to classify endoscopic healing vs. no healing (based on the Mayo endoscopic subscore ≤1)		
Statistic	Value	95% CI
Sensitivity	84.62%	54.55% to 98.08%
Specificity	100.00%	63.06% to 100.00%
Positive Likelihood Ratio		
Negative Likelihood Ratio	0.15	0.04 to 0.55
Disease prevalence	61.90%	38.44% to 81.89%
Positive Predictive Value	100.00%	71.51% to 100.00%
Negative Predictive Value	80.00%	52.78% to 93.47%
Accuracy	90.48%	69.62% to 98.83%
3B. Statistic metrics of a sigmoid BWT cut-off of ≥3.6mm at week 8 to classify clinical response vs. no response (based on decreases in the full Mayo score ≥3)		
Statistic	Value	95% CI
Sensitivity	90.00%	55.50% to 99.75%
Specificity	81.82%	48.22% to 97.72%
Positive Likelihood Ratio	4.95	1.39 to 17.64
Negative Likelihood Ratio	0.12	0.02 to 0.80
Disease prevalence	47.62%	25.71% to 70.22%
Positive Predictive Value	81.82%	55.81% to 94.13%
Negative Predictive Value	90.00%	57.86% to 98.33%
Accuracy	85.71%	63.66% to 96.95%

3 A - to classify endoscopic healing vs. no healing (based on the EMS ≤ 1).

3B - to classify clinical response vs. no response (based on decreases in the fMS ≥ 3).

In our cohort, an MUC of 6.34 at week 8 yields a sensitivity of 76.9% and a specificity of 87.5%.to classify endoscopic healing. The best MUC cut-off at week 8 was a score of 4.9, yielding a sensitivity of 100% and specificity of 75% (area under the curve [AUC] 0.923, [95% CI: 0.805–1.041]), *p* < 0.001). The best MUC cut-off at week 8 to classify clinical response (a decrease in the fMS ≥ 3) was a score of 6.55, yielding a sensitivity of 80% and specificity of 81.8% (area under the curve [AUC] 0.850, [95% CI: 0.677–1.023]), *p* < 0.001).

### Safety outcomes

No new safety outcomes were seen during our induction study, with one patient developing localized herpes zoster, which resolved spontaneously, and the patient continued tofacitinib treatment. Another patient developed urticaria, which was thought unrelated to the study drug.

## Discussion

In this prospective real-world pragmatic study, we evaluated the efficacy of tofacitinib among patients with moderate-severe UC by assessing multiple disease activity endpoints, from clinical to mucosal and transmural responses. Our results demonstrated robust improvements in all aspects of disease activity within the induction period (8 weeks), and we did not reveal any new safety concerns within that period. We also demonstrated that IUS, a single assessment at the end of the induction, is a highly accurate method for detecting treatment response and reflects real-life changes in disease state.

Tofacitinib showed effectiveness and safety in a large RCT for UC, but RCTs’ limitations necessitate real-world evidence (RWE) studies to validate results for broader unrestricted populations. Specifically, pragmatic studies that reflect real-world practice are lacking. Our study prospectively enrolled all patients prescribed tofacitinib by the treating physician, unlike previous prospective trials with stringent inclusion and exclusion criteria. A further strength lies in the spectrum of endpoints we assessed, which included clinical, biochemical, mucosal, histologic, and transmural measures within a real-life longitudinal cohort with stringent follow-up. This comprehensive assessment strategy distinguishes our study from previous real-world studies of tofacitinib effectiveness, which were predominantly retrospective cohort studies or registry-based investigations that evaluated mainly clinical and endoscopic responses to therapy.

Despite our more refractory and unselected patient cohort, our findings closely resembled those in pivotal RCTs and previous RWE studies [4–13]. Our investigation is among the first to report transmural response rates to tofacitinib. Our results indicate that tofacitinib can effectively promote transmural response and healing in many patients within the induction period (8 weeks).

In this study, we demonstrated that IUS is a highly accurate method for detecting treatment response and change in disease state. Specifically, sBWT was a reliable parameter that reflects response to treatment as early as week 8 of treatment induction with a good correlation to endoscopy and clinical response. This aligns with the findings of a previous prospective study examining ultrasonographic evaluation of the colon in patients treated with tofacitinib [21].

Surprisingly, no correlation was found between sBWT and clinical and endoscopic activity at week 0. One possible explanation is the fact that 60% of patients were treated with corticosteroids at week 0, possibly altering IUS features. Also, all of these patients, except one, were transitioned from tofacitinib after failing other therapies; therefore, they were probably partially treated at baseline. Nevertheless, a simple IUS parameter at week 8 – sBWT, could discriminate responders to tofacitinib from non-responders, emerging as an easy-to-use, non-invasive, bed-side tool to assess early response to treatment and aid clinical decisions, perhaps replacing endoscopy. This tool could potentially impact the decision to either decrease tofacitinib dosage at week 8 for responders or prolong the induction phase for an additional 8 weeks.

We used the MUC score on our cohort, designed to evaluate UC endoscopic activity. We found that this score had a good correlation with endoscopic activity at week 8, validating previous studies’ findings [15,20], and we also found a good correlation with the fMS, which, to our knowledge, was not shown in previous studies. Notably, the optimal cut-off in our study to discriminate between active and inactive endoscopic disease differed from the cut-off found in previous studies, perhaps due to the different cohort and population (very experienced patients with prior treatment failures). Still, the cut-off previously described of MUC <6.3, also performed quite well on our cohort.

Of note, histologic change did not occur during the 8-week induction period, similar to previously published trials, and it is plausible that it will happen later [22]. In addition to IUS, we also showed that fecal calprotectin significantly decreased. Faecal calprotectin is a non-invasive alternative for treatment evaluation that is already widely used in managing UC. However, IUS additionally allows evaluation of segmental treatment response and could guide further treatment decision-making. In real-life clinical practice, combining clinical indices, faecal calprotectin, and IUS is likely sufficient to evaluate treatment response for most patients, allowing a more non-invasive but objective approach.

Limitations include a small cohort size and an eight-week assessment time point, which may not be sufficient for evaluating certain treatment targets, the single centre setting, and the single ultrasound examiner. Future studies with larger samples and longer durations are needed. Another limitation is the concomitant treatment with corticosteroids in a significant number of patients at tofacitinib treatment start, thus limiting the ability to differentiate between response to tofacitinib and response to steroids, although at week 8.

In conclusion, our real-world pragmatic study supports the effectiveness of tofacitinib in moderate-severe UC, even after biologic failures, and highlights the potential of IUS for less invasive assessment and monitoring in this population.

## Data Availability

The data underlying this article cannot be shared publicly due to the privacy of individuals who participated in the study. The data will be shared upon reasonable request to the corresponding author.

## References

[1] Feagan BG, Rutgeerts P, Sands BE, et al. Vedolizumab as induction and maintenance therapy for ulcerative colitis. N Engl J Med. 2013;369(8):1–10. doi: 10.1056/NEJMoa1215734. 23964932

[2] Reinisch W, Sandborn WJ, Rutgeerts P, et al. Long-term infliximab maintenance therapy for ulcerative colitis: the ACT-1 and -2 extension studies. Inflamm Bowel Dis. 2012;18(2):201–211. 21484965 10.1002/ibd.21697

[3] Rubin DT, Ananthakrishnan AN, Siegel CA, et al. ACG clinical guideline: ulcerative colitis in adults. Am J Gastroenterol. 2019;114(3):384–413. doi: 10.14309/ajg.0000000000000152. 30840605

[4] Sandborn WJ, Su C, Sands BE, et al. Tofacitinib as induction and maintenance therapy for ulcerative colitis. N Engl J Med. 2017;376(18):1723–1736. doi: 10.1056/NEJMoa1606910. 28467869

[5] Avni-Biron I, Bar-Gil Shitrit A, Koslowsky B, et al. Short-term effectiveness and safety of tofacitinib in ulcerative colitis - real world data from tertiary medical centers in Israel. Dig Liver Dis. 2022;54(2):192–197. 34887214 10.1016/j.dld.2021.11.009

[6] Chaparro M, Garre A, Mesonero F, et al. Tofacitinib in ulcerative colitis: real-world evidence from the ENEIDA registry. J Crohns Colitis. 2021;15(1):35–42. doi: 10.1093/ecco-jcc/jjaa145. 32969471

[7] Biemans VBC, Sleutjes JAM, de Vries AC, et al. Tofacitinib for ulcerative colitis: results of the prospective Dutch initiative on Crohn and colitis (ICC) registry. Aliment Pharmacol Ther. 2020;51(9):880–888. doi: 10.1111/apt.15689. 32237087 PMC7187329

[8] Deepak P, Alayo QA, Khatiwada A, et al. Safety of tofacitinib in a real-world cohort of patients with ulcerative colitis. Clin Gastroenterol Hepatol. 2021;19(8):1592–1601. 32629130 10.1016/j.cgh.2020.06.050 PMC7779667

[9] Hoffmann P, Globig AM, Thomann AK, et al. Tofacitinib in treatment-Refractory moderate to severe ulcerative colitis: real-world experience from a retrospective multicenter observational study. J Clin Med. 2020;9(7):2177. 10.3390/jcm9072177 PMC7408885 32664204

[10] Honap S, Chee D, Chapman TP, et al. Real-world effectiveness of tofacitinib for moderate to severe ulcerative colitis: a multicentre UK experience. J Crohns Colitis. 2020;14(10):1385–1393. doi: 10.1093/ecco-jcc/jjaa075. 32280965

[11] Lair-Mehiri L, Stefanescu C, Vaysse T, et al. Real-world evidence of tofacitinib effectiveness and safety in patients with refractory ulcerative colitis. Dig Liver Dis. 2020;52(3):268–273. 31732444 10.1016/j.dld.2019.10.003

[12] Lucaciu LA, Constantine-Cooke N, Plevris N, et al. Real-world experience with tofacitinib in ulcerative colitis: a systematic review and meta-analysis. Therap Adv Gastroenterol. 2021;14:17562848211064004. doi: 10.1177/17562848211064004. PMC8721385 34987608

[13] Weisshof R, Aharoni Golan M, Sossenheimer PH, et al. Real-World experience with tofacitinib in IBD at a tertiary center. Dig Dis Sci. 2019;64(7):1945–1951. doi: 10.1007/s10620-019-05492-y. 30734234 PMC6935176

[14] Buisson A, Gonzalez F, Poullenot F, et al. Comparative acceptability and perceived clinical utility of monitoring tools: a nationwide survey of patients with inflammatory bowel disease. Inflamm Bowel Dis. 2017;23(8):1425–1433. 28570431 10.1097/MIB.0000000000001140

[15] Allocca M, Fiorino G, Bonovas S, et al. Accuracy of humanitas ultrasound criteria in assessing disease activity and severity in ulcerative colitis: a prospective study. J Crohns Colitis. 2018;12(12):1385–1391. doi: 10.1093/ecco-jcc/jjy107. 30085066 PMC6260119

[16] Maaser C, Petersen F, Helwig U, et al. Intestinal ultrasound for monitoring therapeutic response in patients with ulcerative colitis: results from the TRUST&UC study. Gut. 2020;69(9):1629–1636. doi: 10.1136/gutjnl-2019-319451. 31862811 PMC7456734

[17] Lewis JD, Chuai S, Nessel L, et al. Use of the noninvasive components of the Mayo score to assess clinical response in ulcerative colitis. Inflamm Bowel Dis. 2008;14(12):1660–1666. 18623174 10.1002/ibd.20520 PMC2597552

[18] Marchal-Bressenot A, Salleron J, Boulagnon-Rombi C, et al. Development and validation of the nancy histological index for UC. Gut. 2017;66(1):43–49. doi: 10.1136/gutjnl-2015-310187. 26464414

[19] Limberg B. Diagnosis of chronic inflammatory bowel disease by ultrasonography. Z Gastroenterol. 1999;37(6):495–508. 10427656

[20] Allocca M, Filippi E, Costantino A, et al. Milan ultrasound criteria are accurate in assessing disease activity in ulcerative colitis: external validation. United European Gastroenterol J. 2021;9(4):438–442. 10.1177/2050640620980203 PMC8259285 33349199

[21] de Voogd F, van Wassenaer EA, Mookhoek A, et al. Intestinal ultrasound is accurate to determine endoscopic response and remission in patients with moderate to severe ulcerative colitis: a longitudinal prospective cohort study. Gastroenterology. 2022;163(6):1569–1581. doi: 10.1053/j.gastro.2022.08.038. 36030056

[22] Ollech JE, Yanai H, Avni-Biron I, et al. Fecal calprotectin and quality of life questionnaires are responsive to change in pouch disease activity after antibiotic therapy: results from a prospective clinical trial. Am J Gastroenterol. 2023;118(2):367–370. doi: 10.14309/ajg.0000000000002038. 36191275

